# Comparison of VILIP-1 and VILIP-3 Binding to Phospholipid Monolayers

**DOI:** 10.1371/journal.pone.0093948

**Published:** 2014-04-03

**Authors:** Samuel Rebaud, Anne Simon, Conan K. Wang, Lyndel Mason, Loïc Blum, Andreas Hofmann, Agnès Girard-Egrot

**Affiliations:** 1 Institut de Chimie et Biochimie Moléculaires et Supramoléculaires, Université Lyon 1, University of Lyon, ICBMS, CNRS UMR 5246, Bât. Curien, 43 bd du 11 Nov. 1918, F-69622 Villeurbanne cedex, France; 2 Structural Chemistry Program, Eskitis Institute, Griffith University, Brisbane, Queensland, Australia; University of Oldenburg, Germany

## Abstract

The neuronal calcium sensor proteins Visinin-like Proteins 1 (VILIP-1) and 3 (VILIP-3) are effectors of guanylyl cyclase and acetyl choline receptors, and transduce calcium signals in the brain. The “calcium-myristoyl” switch, which involves a post-translationally added myristoyl moiety and calcium binding, is thought to regulate their membrane binding capacity and therefore, play a critical role in their mechanism of action. In the present study, we investigated the effect of membrane composition and solvent conditions on the membrane binding mechanisms of both VILIPs using lipid monolayers at the air/buffer interface. Results based on comparison of the adsorption kinetics of the myristoylated and non-myristoylated proteins confirm the pivotal role of calcium and the exposed myristol moiety for sustaining the membrane-bound state of both VILIPs. However, we also observed binding of both VILIP proteins in the absence of calcium and/or myristoyl conjugation. We propose a two-stage membrane binding mechanism for VILIP-1 and VILIP-3 whereby the proteins are initially attracted to the membrane surface by electrostatic interactions and possibly by specific interactions with highly negatively charged lipids head groups. The extrusion of the conjugated myristoyl group, and the subsequent anchoring in the membrane constitutes the second stage of the binding mechanism, and ensures the sustained membrane-bound form of these proteins.

## Introduction

The group of Visinin-Like Proteins (VILIPs), a subfamily of Neuronal Calcium Sensor (NCS) Proteins, consists of VILIP-1, VILIP-2, VILIP-3, hippocalcin and neurocalcin δ, all of which bind to cellular membranes. Although their precise functions are still unknown, VILIPs have been related to many membrane-binding properties, such as signal transduction pathways, neurotransmitter release, modulation of ion channel function, and regulation of gene expression [1], [2]. Recently, VILIPs have also been implicated in neuropathological processes ranging from Alzheimer's disease to schizophrenia, and cancer [3]–[6].

Structurally, all VILIPs are characterized by four EF-hand motifs which harbor calcium binding motifs. The amino-acid sequences of VILIPs are highly conserved and the very N-terminal sequence is composed of a consensus sequence (M-G-(X)_3_-S) which harbors a myristoylation site. VILIP-1 and VILIP-3 share about 67% amino acid identity. The membrane association of VILIP-1 [7] and VILIP-3 [8] is thought to be regulated by the “calcium-myristoyl switch” [9], a mechanism that was first characterized in details for recoverin, the prototypic member of the NCS family [10]. For recoverin, the N-terminally conjugated myristoyl group, which is sequestered in a hydrophobic pocket in the absence of calcium, is exposed when the protein undergoes a conformational change due to calcium binding, resulting in insertion of the myristoyl moiety into a membrane.

Despite these similarities, VILIP-1 and VILIP-3 appear to act in different cellular pathways: VILIP-1 has been identified as a modulator of nicotinic acetylcholine receptor [11], as well as the guanylyl cyclase receptor [12]. Sallese *et al*. found *in vitro* that VILIP-1 influences the activity of G-protein coupled receptor kinases 1 and 2 [13], and other cell signaling processes [14]. VILIP-3, in contrast, has been suggested to affect the MAP kinase signaling [15].

In humans, VILIP-1 and VILIP-3 are expressed in cells of the retina and in the brain. VILIP-1 is strongly expressed in cerebellar granule cells, and VILIP-3 is found in Purkinje cells of the cerebellum [8], [16], [17]. The translocation of these proteins from the cytosol to the membrane and their reversible localization to distinct membrane compartments [17], [18] is generally believed to constitute the mechanistic implication of VILIPs in signal transduction [15].

It has previously been found that VILIP-1 and VILIP-3 bind to membranes with varying degrees and/or mechanisms of calcium-dependent membrane association. For instance, in cerebellar extracts, and in the presence of calcium, VILIP-1 and VILIP-3 are found in the membrane fractions. EGTA extraction of the membrane fraction yields less release of VILIP-1 than of VILIP-3. VILIP-3 binding to membranes appears to be less strong than for VILIP-1 [15]. VILIP-1 and VILIP-3 differ in their calcium affinities [19], [20].

In the present study, we attempt a comparative analysis of VILIP-1 and VILIP-3 membrane binding properties in their unmodified as well as post-translationally modified forms, by using model membranes.

Model membranes such as lipid monolayers at the air/water interface [21] have been extensively used to characterize protein interactions with membranes [22], [23]. The advantage of such model systems is that they do not require additional functionalization (for example, by fluorescent groups) that may present steric restraints. Additionally, physical membrane properties such as the packing of lipid molecules can be controlled. For these reasons, lipid monolayer models were chosen in this work.

Since it has been established earlier that the binding of VILIPs to phospholipid membranes involves polar and electrostatic attractions between protein and membrane [24], [25], most likely mediated through several highly conserved lysine residues in the N-terminal region [24], the model membrane systems used in this study were chosen to include zwitterionic as well as negatively charged lipids.

## Materials and Methods

### Materials

Ultrapure water (resistivity: 18.2 M
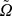
cm) was obtained from an ELGA purelab option Q7 system (VEOLIA WATER STI, France). 4-(2-hydroxyethyl)-1-piperazineethanesulfonic acid (HEPES), sodium chloride (NaCl) and calcium chloride (CaCl_2_) were purchased from Sigma (Saint Quentin Fallavier, France). Ethylenediaminetetraacetic acid (EDTA) was from Chimie Plus Laboratoire (Denice, France). 1,2-Dioleoyl-sn-glycero- 3-phosphocholine (DOPC), 1,2-dioleoyl-sn-glycero- 3-phosphoserine (DOPS), 1,2-dimyristoyl-sn-glycero- 3-phosphocholine (DMPC) and 1,2-dimyristoyl-sn-glycero- 3-phosphoserine (DMPS) were purchased from Avanti Polar Lipids (Coger, France). The phospholipid solutions were prepared by mixing the desired amount of DMPS/DMPC or DOPS/DOPC at 3:1 or 1:3 molar ratio dissolved in chloroform solution at a concentration of 1 mg/mL.

### Methods

#### Preparation of recombinant VILIPs

Bacterial expression plasmids for human VILIP-1 in pET8c [26] and VILIP-3 in pRSET_C were transformed into competent *E. coli* BL21(DE3) cells.

The expression of non-myristoylated proteins followed an in-house adaptation of the auto-induction protocol described by Studier [27]. A total of 8 L of LB auto-induction medium (0.1 mg.L^−1^ ampicillin) were inoculated with an overnight culture of 1 L. The cells were grown at 37°C for 4 hours; incubation was then continued over night at 30°C.

For production of myristoylated VILIPs (myr-VILIPs), BL21(DE3) cells were co-transformed with the above VILIP expression plasmids as well as yeast N-myristoyltransferase (NMT) in the vector pmon5839 [28]. Cells were grown in a total of 2 L of LB medium (0.1 mg L^−1^ ampicillin, 0.05 mg L^−1^ kanamycin) at 37°C until the optical density (measured at 600 nm) of the cell culture reached 0.6. Myristic acid was added to a final concentration of 0.2 mM and the culture was left to incubate for 0.5 hr. Expression was induced by adding isopropyl β-D-1-thiogalactopyranoside (IPTG) to the cell culture at a final concentration of 0.25 mM and the cells were grown at 25°C for 16 hr before harvesting.

After harvest, the bacterial cells were resuspended (100 mM NaCl, 1 mM EDTA, 20 mM TRIS (pH 8), 0.1% Triton X-100, 1 mM PMSF, 5 mM benzamidinium chloride), and lysed by multiple freeze-thaw cycles and subsequent sonication. The resulting suspension was cleared by ultracentrifugation (100000 g, 30 min, 4°C). The supernatant from the ultracentrifugation step was then dialysed against 20 mM TRIS (pH 8), and subjected to anion exchange chromatography using a QA52 column and a gradient of 0–1 M NaCl in 20 mM TRIS (pH 8). Appropriate fractions were pooled and dialysed against 100 mM NaCl, 1 mM MgCl_2_, 1 mM CaCl_2_, 0.1 mM dithiothreitol (DTT) and 20 mM HEPES (pH 7.5). The dialysed sample was then further purified by hydrophobic interaction chromatography using a phenyl sepharose column and isocratic elution with a buffer consisting of 100 mM NaCl, 2 mM EDTA, 0.1 mM DTT, 20 mM HEPES (pH 7.5). After pooling appropriate fractions, the protein sample was concentrated and the buffer exchanged to 100 mM NaCl, 20 mM HEPES (pH 7.5). Protein quality was monitored throughout all purification procedures using denaturing SDS-PAGE.

#### Monolayer Adsorption

Surface pressure measurements were monitored by using a Wilhelmy plate at 20°C and a computer-controlled Langmuir film balance (KSV/NIMA Finland). Langmuir films were made either in a circular Teflon trough containing 30 mL of filtered buffer for protein/lipid interaction analysis or in a rectangular Teflon trough with an area of 14 cm×3.5 cm×0.4 cm and moveable barriers for Maximum Insertion Pressure (MIP) measurements.

In both cases, the subphase buffer consisted of 20 mM HEPES (pH 7.4), 150 mM NaCl, and either 2 mM CaCl_2_ or 2 mM EDTA, as mentioned in the text. Few microliters of a phospholipid solution in chloroform were spread using a Hamilton syringe onto the buffer subphase until the desired initial surface pressure was reached. After the solvent has evaporated and the lipid monolayer has reached equilibrium surface pressure (∏_i_), the protein solution was injected with a Hamilton syringe into the subphase through the lipid monolayer, under gentle stirring. The optimal final concentration of 30 nM protein has previously been optimized [24]. The surface pressure (∏) was recorded continuously as a function of time until a new equilibrium surface pressure (∏_e_) was reached. The increased surface pressure Δ∏ (in mN/m) corresponding to ∏_e_ - ∏_i_ is ascribed to the specific protein interaction with the monolayer. Experiments were repeated at different initial surface pressures ∏_i_. For each experiment, a new Langmuir film was prepared at a given initial pressure (∏_i_) and surface pressure changes (Δ∏) were monitored. After injection of protein, the equilibrium surface pressure (∏_e_) was determined for the different values of the initial surface pressure (∏_i_) of the lipid monolayer. All measurements were repeated between two and five times for each condition.

#### Analysis of surface pressure vs. time measurements

The curve of measured surface pressure (∏) as a function of time after VILIP injection into the subphase underneath the monolayer was analyzed as followed: the surface pressure variation (Δ∏) corresponding to ∏_e_ - ∏_i_, and the tangent to the curve at the origin (time of protein injection) corresponding to the initial rate of binding to lipid monolayer, were determined for the different value of ∏_i_. The relative kinetics of proteins binding was calculated as a percentage of increased initial rates of binding in the presence of calcium relative to those in the absence of calcium.

The curve of surface pressure changes (Δ∏) vs. initial surface pressure (∏_i_) allowed determination of the MIP by extrapolation of the data to the x-axis [29]. The Synergy factor (*a*) corresponds to the slope of the linear regression of equilibrium surface pressure (∏_e_) plotted vs. the initial surface pressure (∏_i_) [29].

## Results

### Adsorption of VILIPs on membranes

**Figure 1:**
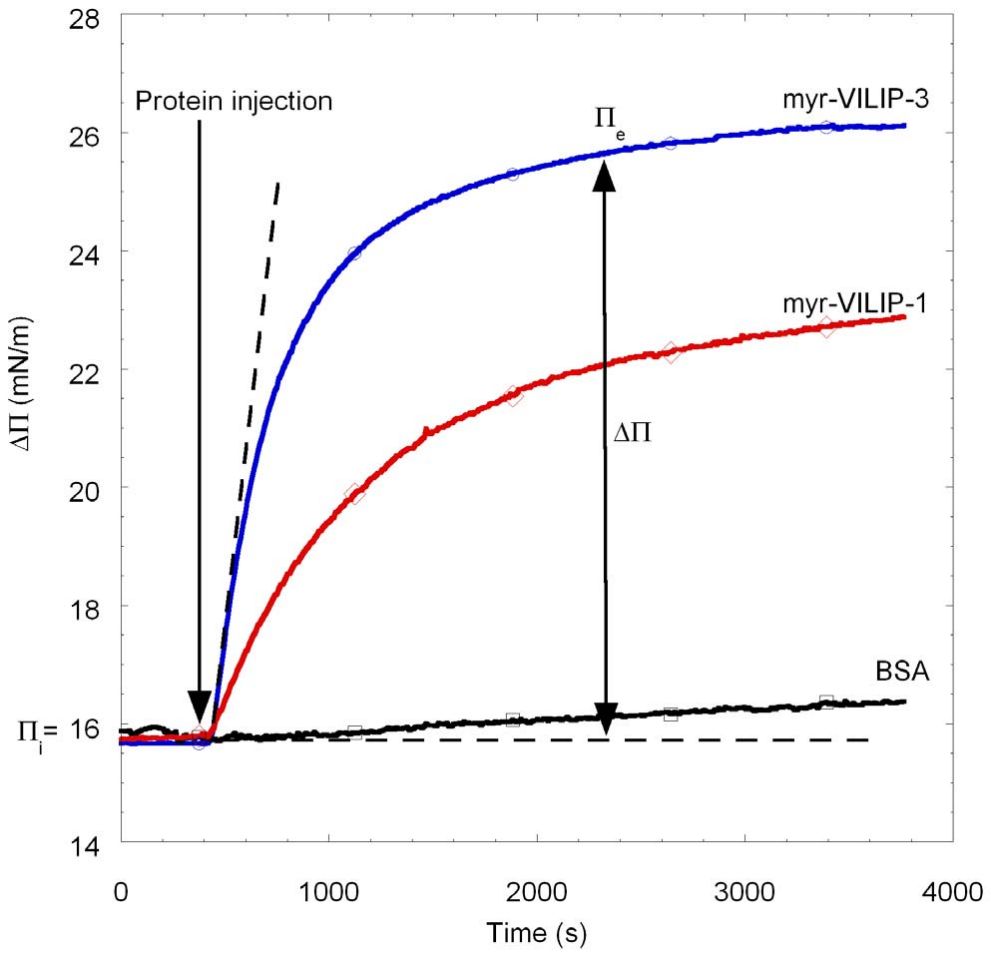
Adsorption of myristoylated VILIPs and BSA to phospholipid monolayers. Surface pressure (Δ∏) change vs. time after injection of myr-VILIP-1, myr-VILIP-3 or Bovine Serum Albumine (BSA) into subphase beneath phospholipid monolayers of DOPS/DOPC (at molar ratio 3:1) compressed at an initial surface pressure (∏i) of about 16 mN/m. The final concentration of myr-VILIP-1 (red,○?), myr-VILIP-3 (blue, ⋄), and BSA (black, □) was 30 nM. The subphase consisted of 10 mM HEPES at pH 7.4, 150 mM NaCl, 2 mM CaCl2. After proteins injection (indicated by an arrow), the surface pressure increased until saturation at the equilibrium adsorption pressure (∏_e_). Note that the dotted line is the tangent to the curve at initial time of protein injection and corresponds to the initial rate of binding to lipid monolayer.

The first goal was to confirm the membrane binding properties of VILIP-1 and -3 versus the phospholipid composition of the monolayer and versus the presence of calcium ions in the bulk. Figure 1 shows a representative measurement of surface pressure difference (Δ∏in mN/m) as a function of time after myr-VILIP-3 injection into the subphase underneath DOPS/DOPC (at molar ratio 3:1) monolayer. This result is representative of all membrane association kinetics of VILIP-1 and VILIP-3 both in their myristoylated (myr-VILIPs) and non-myristoylated (VILIPs) forms. For both VILIP proteins, myr-VILIP-1 and myr-VILIP-3, an immediate increase of the surface pressure was observed upon protein injection. In contrast, in control experiments using bovine serum albumin (BSA), no interaction of BSA with the Langmuir films was observed (Figure 1). In all measurements involving VILIPs, the surface pressure increase follows a Langmuir-type saturation curve and finally reaches an equilibrium adsorption pressure value (∏_e_). The difference between the initial and the equilibrium surface pressure constitutes a characteristic surface pressure difference Δ∏ variation for an individual protein. The surface pressure difference is thus a measure for the interactions of VILIPs with the Langmuir monolayer. Irrespective of the presence or absence of calcium, membrane composition or use of myristoylated or non-myristoylated proteins, no major qualitative difference in Δ∏ changes over time was observed. The tested VILIP-1 and VILIP-3 proteins bind to model membranes under all experimental conditions used in this study. We thus decided to undertake a quantitative analysis of the influence of each parameter is of interest.

### Dependence on lipid composition of monolayers

Recent works have demonstrated that non-myristoylated VILIP-1 binds to DMPS/DMPC (3:1) monolayers in the absence of calcium [24] and that myristoylated VILIP-1 and non-myristoylated VILIP-1 bind to DOPS/DOPC (3:1) monolayers in the presence and absence of calcium [30]. The results of Braunewell *et al*. [24] with lipid monolayers containing phosphatidylinositol phosphate (PIP) derivates as well as with cells support the idea of specific interactions of VILIPs with PIPs and electrostatic interactions. The effect of lipid composition on the membrane binding properties of VILIP-1 and VILIP-3 was investigated in terms of fatty acid content and phospholipid head group charge.

### Effect of phospholipid saturation on VILIP binding

The binding of VILIPs to lipid monolayers made of saturated lipid mixtures (DMPS/DMPC) or unsaturated lipid mixtures (DOPS/DOPC), both at a molar ratio of 3:1, was investigated in the presence or absence of calcium.

**Figure 2:**
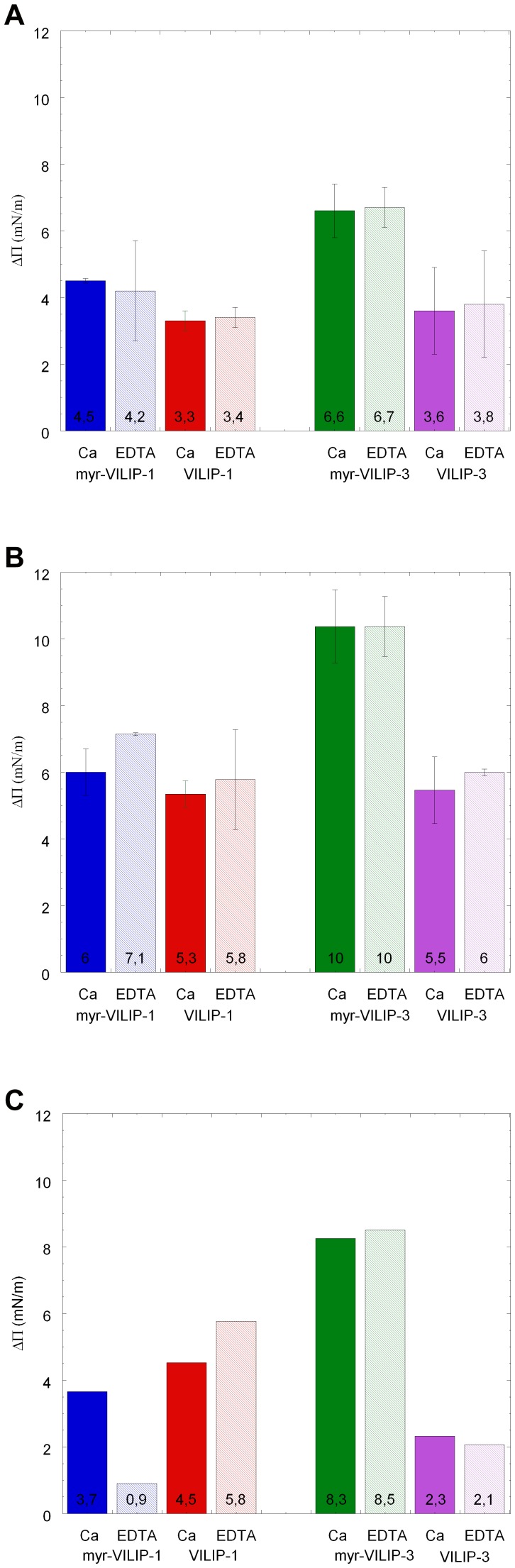
Adsorption of VILIPs to monolayers of different lipid compositions under varying buffer conditions. Histograms of averaged Δ∏ for three different lipid monolayers in the absence or presence of calcium: myr-VILIP-1 (blue), VILIP-1 (red), myr-VILIP-3 (green), and VILIP-3 (purple). The phospholipid monolayers are composed of A) DMPS/DMPC (at molar ratio 3:1), B) DOPS/DOPC (3:1), and C) DOPS/DOPC (1:3) as indicated. The subphase consisted of 10 mM Hepes at pH 7.4, 150 mM NaCl, 2 mM CaCl_2_ or 2 mM EDTA. Conditions in the presence or absence of calcium are shown in full and striped color, respectively. The error bars indicate the standard deviation of between two and five technical repeats.

Figure 2A and Figure 2B shows histograms of averaged Δ∏ measurements in each condition.

Monolayers comprised of unsaturated lipid mixtures (Figure 2B) showed higher Δ∏values than monolayers made of saturated lipid mixtures (Figure 2A), indicating that VILIPs preferentially bind to unsaturated phospholipids. This behavior is similar to that previously reported for recoverin [29], [31].

Moreover, the Δ∏ increase observed during binding of VILIP-1 in its myristoylated and non-myristoylated forms to either DMPS/DMPC (3:1) or DOPS/DOPC (3:1) monolayers is in agreement with previous reports [24], [30].

Our results show also that higher Δ∏ values are obtained for myr-VILIP-1 and myr-VILIP-3 than for the non-myristoylated proteins, which demonstrates the membrane anchoring effect of the myristoyl moiety (in agreement with findings from the analysis of adsorption rates and calcium effects; see below). Independently of the saturation of the fatty acid tails of lipids, Δ∏ values are very similar for the two non-myristoylated proteins.

### Effects of phospholipid charge on VILIP binding

In order to investigate the effects of phospholipid head group charges, monolayer compositions of DOPS/DOPC at molar ratios of 3:1 (Figure 2B) and 1:3 (Figure 2C) were compared. Similar results were obtained for all proteins, except for myr-VILIP-1 which, in the absence of calcium, showed strongly reduced binding to the monolayer with less negative charge. In general, the surface pressure differences observed for VILIP-monolayer adsorption are larger for monolayers with more negative head group charge, i.e. DOPS/DOPC (molar ratio 3:1). This suggests a significant electrostatic component in the VILIP-1 and VILIP-3 interactions with lipid membranes.

### Effect of calcium on VILIP-monolayer interactions

In order to assess the effect of calcium on the binding of VILIPs to Langmuir films, the Δ∏ values obtained for the three different membrane compositions shown in Figures 2A, 2B, and 2C are compared. The first obvious and important finding is VILIPs are able to bind membranes even in the absence of calcium. For three proteins, VILIP-1, myr-VILIP-3, and VILIP-3, a comparison of Δ∏ values for a given composition of lipid monolayer shows no significant difference in the presence (2 mM CaCl_2_) or absence of calcium (2 mM EDTA). This is different for myr-VILIP-1 and the lesser charged monolayer DOPS/DOPC (1:3). Here, a significantly higher Δ∏ value is observed in the presence than in the absence of calcium. Such a behavior would be expected for a calcium-myristoyl switch mechanism.

The comparison of Δ∏ values obtained for myr-VILIP-1 and VILIP-1 binding on DOPS/DOPC (1:3) monolayer reveals that the surface pressure difference is larger for the non-myristoylated protein. This reflects a difference in the extent of protein membrane insertion, and indicates that non-myristoylated VILIP-1 may engage a different binding interface than myr-VILIP-1. In this context, the existence of different mixtures of monomeric and dimeric species of this protein in its conjugated and non-conjugated forms may be of importance [30].

Importantly, no difference in Δ∏ values in the presence or absence of calcium is observed with the higher charged DOPS/DOPC (3:1) monolayer in comparison with DOPS/DOPC (1:3) monolayer, suggesting the existence of an electrostatic binding mechanism.

### Adsorption kinetics

**Figure 3:**
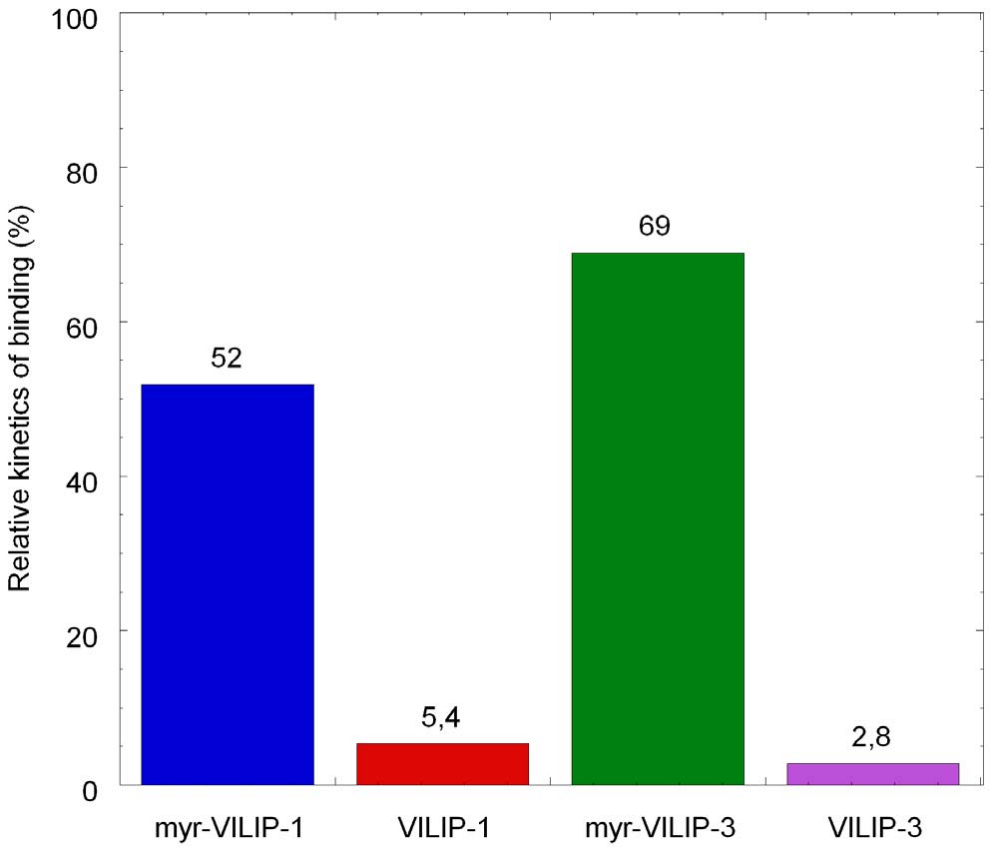
Adsorption kinetics of VILIPs. Histograms of relative kinetics of binding of myristoylated and non-myristoylated VILIPs interaction to DOPS/DOPC (1:3) monolayers in the presence of calcium relative to those in the absence of calcium. Color coding: myr-VILIP-1 (blue), VILIP-1 (red), myr-VILIP-3 (green), and VILIP-3 (purple).

Figure 3 compares the rate constants from binding kinetics of VILIPs in the presence of calcium ions relative to those in the presence of EDTA calculated for myristoylated and non-myristoylated VILIPs. The adsorption is faster for myr-VILIP-1 and myr-VILIP-3 than for non-myristoylated forms. These results clearly demonstrate the importance of the myristoyl group and the calcium ions in buffer as a temporal signal of myr-VILIPs binding. Whereas other factors (e.g. electrostatic interactions) contribute to binding of these proteins to membranes, these results highlight the role of the calcium-myristoyl switch for the kinetics of VILIP-membrane binding.

### Comparison of myr-VILIP-1 and myr-VILIP-3 binding onto lipid monolayers

Of the conditions tested in this study, the adsorption experiments of myristoylated VILIP-1 and -3 to DOPS/DOPC (1:3) monolayer are the most interest when compared with physiological conditions. Analysis of the adsorption data with respect to Maximum Insertion Pressure (MIP), and synergy factor *a* [29], [32], in order to yield further insights into binding mechanisms, is thus carried out for those conditions. The MIP reflects quantity of protein bound to the monolayer and/or extend of protein membrane insertion.

**Figure 4:**
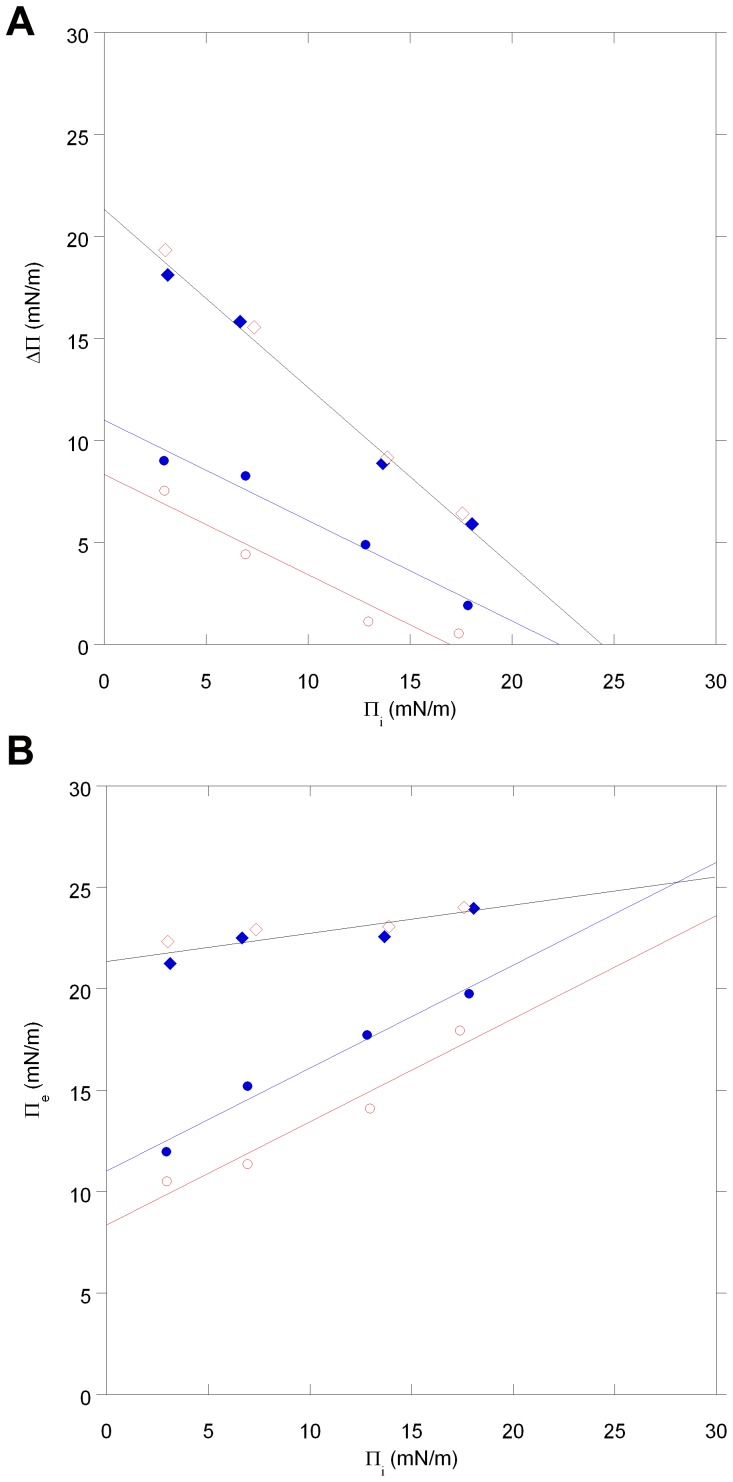
Analysis of binding parameters of myristoylated VILIPs. **A.** Surface pressure changes (Δ∏) vs. initial surface pressure (∏_i_) of myr-VILIP-1 (• and ○) and myr-VILIP-3 (♦ and ⋄) in the presence of calcium (• and ♦) or EDTA (○ and ⋄). MIP values were obtained by extrapolating respectively the curves to the x axis. **B.** Equilibrium adsorption surface pressure (∏_e_) vs. initial surface pressure (∏_i_) of myr-VILIP-1 (• and ○) and myr-VILIP-3 (♦ and ⋄) in the presence of calcium (• and ♦) and EDTA (○ and ⋄). The phospholipid monolayers are composed of DOPS/DOPC (at molar ratio 1:3) and compressed at different initial surface pressures. See also Table S1.

Figure 4A illustrates the linear correlation of the decrease of Δ∏ with the increase of ∏_i_ for the two myristoylated VILIPs. In the case of myr-VILIP-1, the MIP is larger in the presence of calcium ions (MIP: 22.3 mN/m) than in their absence (MIP: 17.0 mN/m), indicating that the binding conditions for myr-VILIP-1 are more favorable in the presence of calcium. With myr-VILIP-3, the plots in the absence and presence of calcium ions are indistinguishable with MIP values of ∼24.6 mN/m. The larger MIP values obtained for myr-VILIP-3 suggests that the quantity of membrane association molecules is larger for myr-VILIP-3 than for myr-VILIP-1. Since the binding of myr-VILIP-3 to cell membranes appears to be less strong than for VILIP-1 [15], we conclude that the larger MIP observed with myr-VILIP-3 indeed indicates the association of more molecules rather than deeper penetration (stronger interaction) of this protein.

The correlation of the equilibrium surface pressure ∏_e_with the initial surface pressure ∏_i_ is illustrated in Figure 4B. Independent of calcium, ∏_e_ increases with an increase of ∏_i_ for both myristoylated proteins. The synergy factor *a*, obtained as the slopes in Figure 4B, is 0.5 for myr-VILIP-1 and 0.1 for myr-VILIP-3. These positive synergy factors indicate favorable conditions for the binding of myristoylated VILIPs to the monolayer. The fact that myr-VILIP-1 possesses a larger synergy factor than myr-VILIP-3 indicates that the physical state and lipid packing of the monolayer has a greater effect on myr-VILIP-1 than myr-VILIP-3 binding. For comparison the same analysis of binding of the unmyristoylated proteins is provided in the Supporting Information (Figure S1, Table S1).

## Discussion

The objective of this study was to compare the membrane mechanisms of VILIP-1 and VILIP-3. So far, it has been generally assumed that all members of the NCS protein family, with recoverin as the prototype, engage the established calcium-myristoyl switch mechanism: upon binding of calcium, the protein undergoes a conformational switch that exposes the myristoyl moiety, which inserts into the membrane and thus provides anchorage in the membrane. Upon photoactivation, which leads to high intra-cellular concentration of calcium, recoverin translocates from the cytosol to the membranes where it binds to rhodopsin kinase and inhibits the rhodopsin phosphorylation [33].

Results obtained in this work show that different members of the NCS protein family exhibit different variations of this mechanism. Comparison of the membrane association of myristoylated and non-myristoylated VILIP-1 and VILIP-3 using Langmuir monolayers has clearly demonstrated the pivotal role of the myristoyl group. This is best illustrated by the binding kinetics (Figure 3) showing significantly lower association rates of the non-myristoylated proteins.

Based on VILIP-3 colocalisation studies in Hippocampal neurons using immunofluorescence, it has previously been concluded that myristoylated VILIP-3 is predominantly cytosolic under non-elevated cytoplasmic calcium concentration [17]. However, there is precedence with similar observed apparent differences in the membrane binding behaviour of NCS proteins. With supported lipid bilayers, no binding of non-myristoylated recoverin to membranes was observed [34], which is in contrast to the findings with lipid monolayers, where binding of both myristoylated and non-myristoylated recoverin was observed [31]. The observed differences between different lipid membrane models, as well as biological membranes in intact cells, might be explained by different membrane parameters (pressure, composition, packing, etc) not all of which can be controlled in the different systems. Importantly, these parameters culminate at the macroscopic level to different local curvatures of lipid membrane systems which can affect protein adsorption behavior [35], and thus account for differing results.

Importantly, when comparing two different proteins in the same lipid membrane system, valid mechanistic insights can be obtained. In the present study, myristoylated VILIP-1 does not bind lipid monolayer in the absence of calcium which is in contrast to the observation made with VILIP-3 (Figure 2C). These findings demonstrate important differences in the mechanism of membrane association between both myristoylated.

When not conjugated with myristic acid, both VILIP-1 and VILIP-3 associate with lipid monolayers, and this binding becomes independent of the presence of calcium. The fact that this adsorption is more pronounced with monolayers that have more negatively charged phospholipid head groups (Figure 2B) suggests that these interactions are mainly of electrostatic nature. This agrees with findings from Surface Plasmon Resonance experiments with recoverin [34], and may be due to the conserved basic residues found in VILIPs [30]. Possible interactions of VILIPs with highly negatively charged phospholipid head groups such as inositol phosphate must be considered. Experimentally, it is well established by PIP strip assay and by immune-fluorescence studies that VILIP-1 can indeed interacts with inositol phosphates [24]. Moreover, the preferential interactions of hippocalcin to phosphatidylinositol phosphates in cell membrane as well as the Golgi localization of this protein indicate specific interactions with inositol phosphate [36], and recognition of specific PIP derivates by a basic cleft in the N-terminal area of these proteins has been proposed [24]. The difference observed in the membrane binding properties of the two VILIP proteins in this study is in agreement with findings by Spilker and coworkers who showed that there is a difference of extractability from membrane fractions between VILIP-1 and VILIP-3 [15].

**Figure 5:**
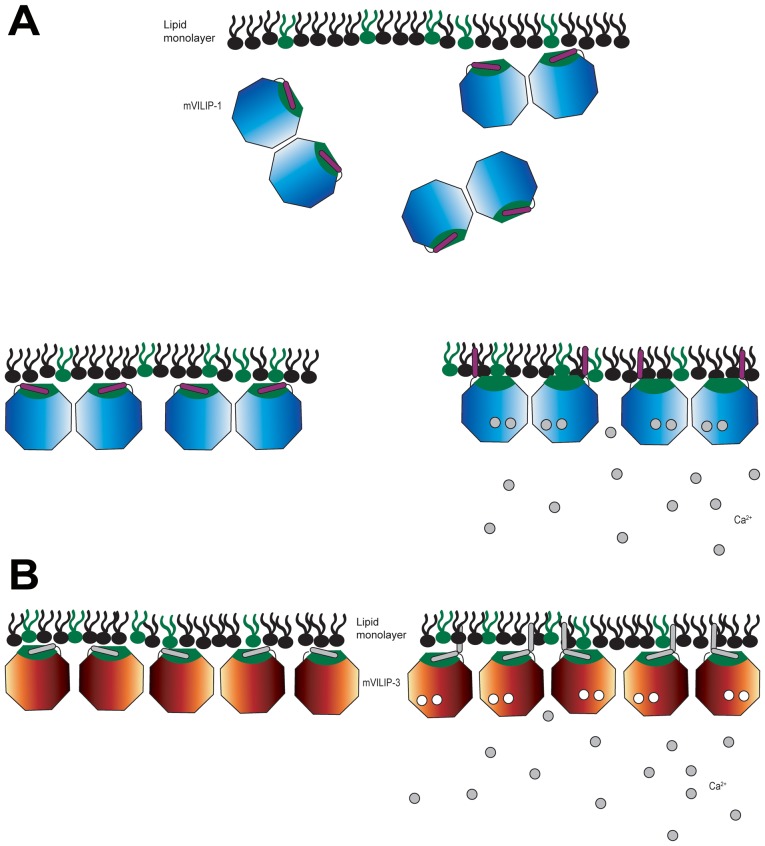
Schematic molecular mechanisms of A) myr-VILIP-1 and B) myr-VILIP-3, in the presence (•) or absence (○) of calcium. The phospholipid monolayers are composed of negatively charged phospholipid head groups (green) and zwitterionic phospholipids (black). VILIP-1 dimers and VILIP-3 monomers bind to membranes by electrostatic interactions, and possibly by specific recognition of highly negatively charged phospholipid head groups such as inositol phosphate. Myr-VILIP-3 is rapidly associated with lipid monolayers in the presence and absence of calcium; the main driving force appears to be electrostatic interactions. Myr-VILIP-1 may display oscillating adsorption and desorption onto lipid monolayers which is decided in favor of adsorption by the presence of calcium and the subsequent exposure and membrane insertion of the myristoyl group. In general, the presence of the myristoyl group enhances the adsorption kinetics and ultimately ensures anchorage of the proteins at the membrane. Not drawn to scale.

Since the presence of the myristoyl conjugation is not required for initial VILIP-1 and -3 association with membranes but significantly affects the binding rates, we propose that the membrane binding mechanism of these proteins consists of two stages, as illustrated in Figure 5. Both VILIP-1 is attracted to the membrane (Figure 5A), mainly by electrostatic interactions with negatively or highly negatively charged phospholipid head groups [24]. At this stage, VILIP-1 molecules may display oscillating adsorption and desorption, and possibly interacting by specific binding with highly negatively charged phospholipid head groups such as inositol phosphate [24]. Ultimately, the initial association is decided in favor of adsorption by extrusion of the myristoyl group which arrests the proteins molecules on the membrane.

In contrast, the initial association of VILIP-3 with membranes is independent of calcium and driven by electrostatic interactions (Figure 5B). Our results also suggest that calcium is not critically required to induce exposure and membrane insertion of the myristoyl group of VILIP-3 (see Figure 4, Table S1). In the case of VILIP-3, the presence of calcium seems to affect solely the binding kinetics (see Figure 3).

The model provided in Figure 5 takes into account observations from VILIP-1 and -3 membrane binding studies with controlled lipid membrane parameters. It is noteworthy that cellular factors may provide further regulation. As it has been pointed out previously, multiple molecular species are possible for VILIPs, since divalent cations and particular Redox conditions can induce dimer formation [30]. Monomeric and dimeric species are certainly to be expected for VILIP-1; however, to date, dimerization of myr-VILIP-3 has not been observed [20], [37]. The temporal and spatial modulation of calcium concentration and subsequent effects on signaling pathways, also need to be considered. The calcium-induced conformational change of VILIP-1 and VILIP-3 may play a role in the activation of a binding partner [30], as also proposed for other calcium-myristoyl switch proteins by Meyer and York [38].

## Conclusion

Findings from this present study indicate the calcium-myristoyl switch of VILIP-1 and VILIP-3 is one, but not the exclusive component of the membrane binding mechanisms of these proteins. Membrane association of these VILIPs appears to be driven by electrostatic interactions, since both myristoylated and non-myristoylated proteins bind to Langmuir monolayers and they do so independently of calcium. With phospholipid monolayers, the presence of calcium affects the kinetics of association of the myristoylated VILIPs with the lipid layer, and sequestration of the myristoyl group serves the purpose of arresting the proteins in the membrane-bound state. Clearly, myristoylated VILIP-1 and VILIP-3 engage different membrane binding mechanisms which indicates that different NCS proteins may engage their individual adaptation of the calcium-myristoyl switch. In case of VILIP-3, calcium appears to be a purely kinetic factor for membrane association.

## Supporting Information

Figure S1 **Comparison of VILIP-1 and VILIP-3 binding in their non-myristylated forms. A.** Surface pressure changes (Δ∏) vs. initial surface pressure (∏_i_) of VILIP-1 (• and ○) and VILIP-3 (♦ and ⋄) in the presence of calcium (• and ♦) and EDTA (○ and ⋄). **B.** Equilibrium adsorption surface pressure (∏_e_) vs. initial surface pressure (∏_i_) of VILIP-1 (• and ○) and VILIP-3 (♦ and ⋄) in the presence of calcium (• and ♦) and EDTA (○ and ⋄). The phospholipid monolayers are composed of DOPS/DOPC (at molar ratio 1:3) and compressed at different initial surface pressures.
(TIF)

Table S1 Comparison of maximum insertion pressures (MIPs) and synergy factors (*a*) for myristoylated and non-myristoylated VILIP-1 and VILIP-3. The phospholipid monolayers were composed of DOPS/DOPC (at molar ratio 1:3).
(DOCX)
