# Supplementary figures and images for: Comparison of VILIP-1 and VILIP-3 Binding to Phospholipid Monolayers
Source: PLoS One. 2014 Apr 3;9(4):e93948. doi: 10.1371/journal.pone.0093948 (PMC3974848; doi:10.1371/journal.pone.0093948)

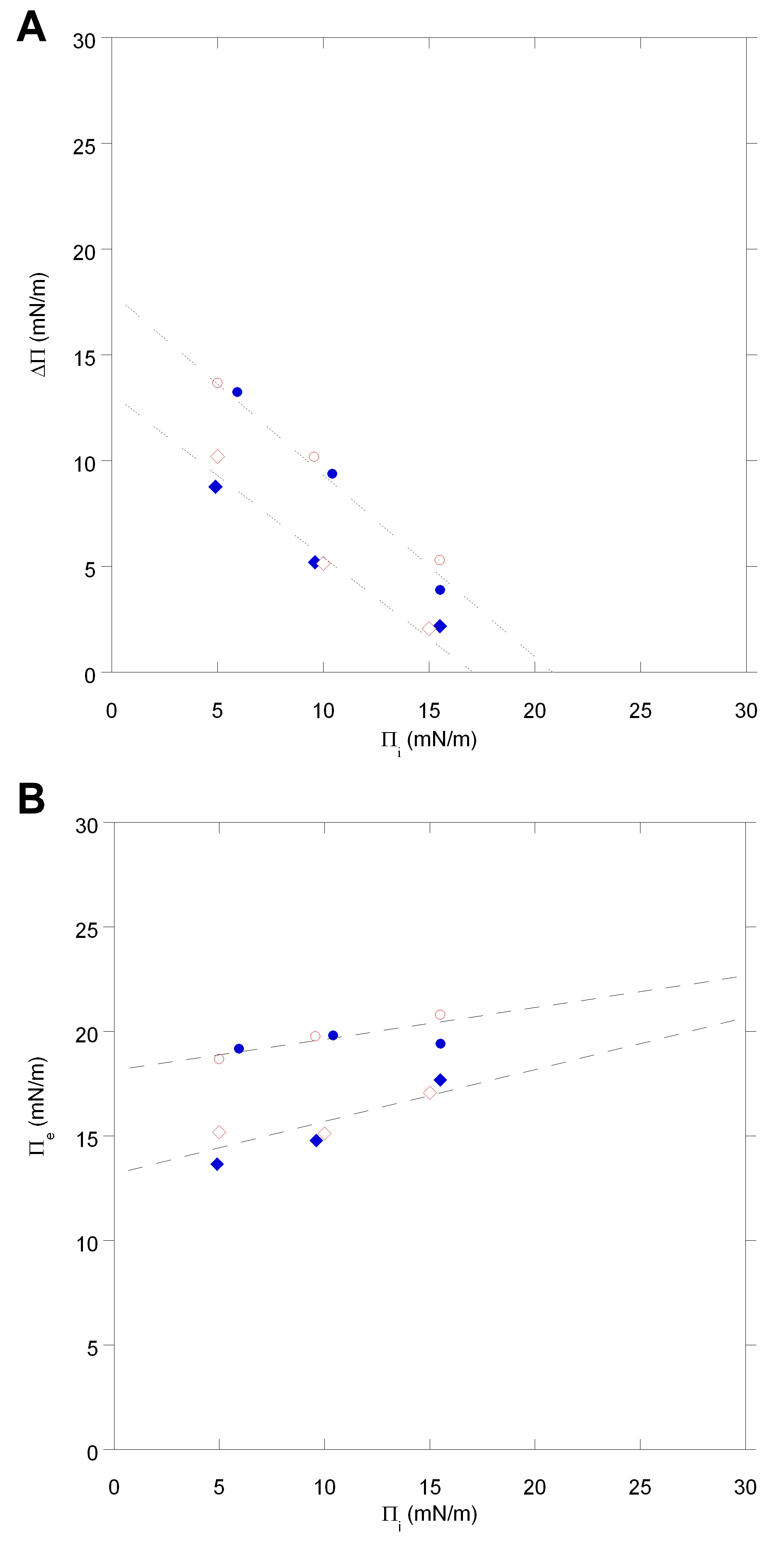

Supplement: Figure S1 — Comparison of VILIP-1 and VILIP-3 binding in their non-myristylated forms. A. Surface pressure changes (Δ∏) vs. initial surface pressure (∏i) of VILIP-1 (• and ○) and VILIP-3 (♦ and ⋄) in the presence of calcium (• and ♦) and EDTA (○ and ⋄). B. Equilibrium adsorption surface pressure (∏e) vs. initial surface pressure (∏i) of VILIP-1 (• and ○) and VILIP-3 (♦ and ⋄) in the presence of calcium (• and ♦) and EDTA (○ and ⋄). The phospholipid monolayers are composed of DOPS/DOPC (at molar ratio 1:3) and compressed at different initial surface pressures. (TIF) [file pone.0093948.s001.tif]
