# Supplementary material for: Comparison of VILIP-1 and VILIP-3 Binding to Phospholipid Monolayers
Source: PLoS One. 2014 Apr 3;9(4):e93948. doi: 10.1371/journal.pone.0093948 (PMC3974848; doi:10.1371/journal.pone.0093948)
Supplement: Table S1 — Comparison of maximum insertion pressures (MIPs) and synergy factors (a) for myristoylated and non-myristoylated VILIP-1 and VILIP-3. The phospholipid monolayers were composed of DOPS/DOPC (at molar ratio 1:3). (DOCX) [file pone.0093948.s002.docx]

|  | MIP (mN/m) | *a* |
| --- | --- | --- |
| ***Absence of calcium*** |  |  |
| VILIP-1 | 20.8 | 0.1 |
| myr-VILIP-1 | 17.0 | 0.5 |
| VILIP-3 | 17.9 | 0.3 |
| myr-VILIP-3 | 24.6 | 0.1 |
| ***Presence of calcium*** |  |  |
| VILIP-1 | 20.8 | 0.1 |
| myr-VILIP-1 | 22.3 | 0.5 |
| VILIP-3 | 17.9 | 0.3 |
| myr-VILIP-3 | 24.6 | 0.1 |
